# Temporal Trend of Gestational Syphilis between 2008 and 2018 in Brazil: Association with Socioeconomic and Health Care Factors

**DOI:** 10.3390/ijerph192416456

**Published:** 2022-12-08

**Authors:** Janmilli da Costa Dantas, Cristiane da Silva Ramos Marinho, Yago Tavares Pinheiro, Richardson Augusto Rosendo da Silva

**Affiliations:** 1 Postgraduate Program in Public Health, Federal University of Rio Grande do Norte, Natal 59064-630, Brazil; 2 Faculty of Health Sciences of Trairi, Federal University of Rio Grande do Norte, Santa Cruz 59078-900, Brazil; 3 Center of Health Sciences, Department of Nursing, Federal University of Rio Grande do Norte, Natal 59078-900, Brazil

**Keywords:** syphilis, prenatal care, pregnancy, communicable diseases, *Treponema pallidum*

## Abstract

The increased number of cases in recent years has turned syphilis into a global public health problem. In 2020, 115,371 cases of acquired syphilis were reported (detection rate of 54.5 cases/100,000 inhabitants) in Brazil. In that same period, the country notified 61,441 cases of gestational syphilis (detection rate of 21.6 per 1000 live births). The number of syphilis cases points to the need to reinforce surveillance, prevention, and infection control actions, which is a worrying scenario for government organizations. This study aims to describe the temporal trend of gestational syphilis from 2008 to 2018 in Brazilian regions and to associate its detection rate with socioeconomic and health care indicators. We conducted an ecological study of temporal trends using secondary data from the Department of Informatics of the Unified Health System. The temporal trend was analyzed using the Joinpoint Regression program. The annual percent change (APC) and 95% confidence intervals (95%CI) were estimated and tested; statistical significance was assessed using the Monte Carlo permutation test. Correlations were assessed using Pearson’s correlation coefficient, and statistical significance was calculated using Pearson’s product-moment correlation. The gestational syphilis detection rate increased between 2008 and 2018. The South region showed the greatest trend, whereas the Midwest region presented the lowest trend. The following variables were significantly correlated with the gestational syphilis detection rate: Municipal Human Development Index, illiteracy rate, percentage of primary health care coverage, and proportion of doctors, nurses, and basic health units per inhabitant. Health policies are needed to mitigate social vulnerabilities and strengthen primary health care.

## 1. Introduction

Syphilis is a curable sexually transmitted infection that causes systemic consequences and is exclusive to humans [1]. It affects the health and lives of people worldwide and impacts reproductive and child health. Syphilis may also cause consequences during pregnancy, such as abortions, stillbirth, premature birth, infant or neonatal death, and early or late congenital manifestations [2].

The increased number of cases in recent years has turned syphilis into a global public health problem, despite protocols for prevention, diagnosis, and treatment. In the world, in 2020, about 7.1 million new cases of syphilis in people between 15 and 49 years of age were reported [3]. Developed countries in North America and Western Europe, which had their incidence of syphilis cases reduced in the 1980s and 1990s, showed an increase from the 2000s onwards [4,5]. The highest incidence rates of syphilis were found in countries in Latin America, Africa, and Asia, in lower income territories [6]. In 2016, more than 900,000 pregnant women worldwide were infected with syphilis [7]. In 2020, cases of gestational syphilis (GS) have increased in several American countries [8].

In Brazil, syphilis reemerged and was declared an epidemic in 2016 [9]. In 2020, Brazil registered 61,441 cases of GS (detection rate of 21.6 per 1000 live births), 22,065 cases of congenital syphilis (detection rate of 7.7 per 1000 live births), and 186 deaths by congenital syphilis [10].

In 2014, the Pan American Health Organization (PAHO) created a committee to analyze the vertical transmission of syphilis and reduce its transmissibility. To validate a country that reached the goal, the PAHO recommends that 95% of pregnant women have access to at least one prenatal consultation and be tested and treated for syphilis; eleven countries were already validated [11]. The United Nations also proposed sustainable development goals, including eliminating congenital syphilis by 2030 and reducing its incidence to 0.5 cases per 1000 live births [12].

In this context, information about the detection rate in the territory is needed for evaluation, planning, and decision-making regarding syphilis control [13]. Thus, we aim to fill gaps in the literature regarding the GS detection rate in five Brazilian macro-regions and its correlations with socioeconomic and health care factors. Our research is relevant because time series analysis may show the evolution of disease incidence, which is suitable for difficult-to-control diseases, such as GS. We also highlight indicators related to the number of human resources and physical structure of the health care network in primary care, which are still little explored in national studies, especially regarding GS. Our study is also important at national and international levels since countries with contexts similar to Brazil aiming to reduce the incidence of GS may consider our results to formulate strategies and health policies.

Therefore, our study aims to describe the temporal trend of GS and associate its detection rate with socioeconomic and health care indicators in all Brazilian regions between 2008 and 2018. 

## 2. Materials and Methods

This is a population-based ecological epidemiological time-series study using secondary public-domain data. Brazil is the largest country in South America, presenting a territorial extension of 8,510,295 km² and an estimated population of 214,326,223 inhabitants (data from 2021). According to the Brazilian Institute of Geography and Statistics, the country comprises five macro-regions (North, Northeast, Midwest, Southeast, and South), 5570 municipalities, 27 federative units, and 1 Federal District [14].

The timeline for obtaining the data was between 2008 and 2018. Data were collected between June and July 2021 from publicly accessible databases linked to the Ministry of Health of Brazil, the Department of Informatics of the Unified Health System (DATASUS), the e-Manager primary care (e-Gestor AB), and the National Human Development Program. Regarding the GS detection rate, the number of reported cases by city of residence was obtained from the Notifiable Diseases Information System (SINAN), whereas the number of live births was obtained from the Information System of Live Births (SINASC). This study was not submitted for ethical consideration because secondary data were in the public domain (resolution nº 510 of the National Health Council).

The health information systems in Brazil were created from the 1970s onwards, with the information system on live births (SINASC) being the oldest, and one of those that presents the most reliable information, therefore, of better quality. This fact is due to a policy developed by the Ministry of Health for the mandatory registration of the newborn, which is conditioned to the completion of a declaration that is used to feed the SINASC. However, the country still faces problems of underreporting, mainly in relation to the feeding of data in the other systems implanted more recently. The lack of completeness of the data was observed in some information systems by some municipalities.

Brazil is composed of five macro-regions; however, due to its large territory, it is possible to observe a social inequality between its regions that results in the development of each one. This scenario directly reflects the supply of health information systems. Studies indicate that more developed regions have less data underreporting.

The GS detection rate was obtained by dividing the number of reported cases in a given region and the period by the number of live births in a given region and period, multiplied by 1000. The Brazilian Ministry of Health [15] adopted the following criteria for notifying GS: asymptomatic women without previous treatments and with at least one reagent test (treponemal or non-treponemal with any titration or both) during prenatal care, childbirth, or puerperium; symptomatic women with at least one reagent test (treponemal or non-treponemal with any titration or both) during prenatal care, childbirth, or puerperium; or women with at least one reagent test (treponemal or non-treponemal with any titration or both) during prenatal care, childbirth, or puerperium, regardless of symptoms and previous treatments.

To prevent vertical transmission of syphilis, the Brazilian Ministry of Health includes testing for syphilis in pregnant women as routine prenatal care. The guidelines for screening syphilis in pregnant women consist of performing 3 tests. Testing should be performed at the first prenatal visit, repeated in the third trimester of pregnancy, and at the time of admission for delivery using a rapid test or serology [1,16].

The following independent variables were included: health care variables related to human resources in health services (proportion of primary care physicians and nurses per inhabitant—obtained from the National Registry of Health Facilities); number of physical health care structures (proportion of primary health care unit per inhabitant—obtained from National Registry of Health Facilities); access to prenatal care (percentage coverage of primary care teams—consulted in the e-Gestor AB database); percentage of live births without prenatal consultation, percentage of live births with 1 to 3 prenatal consultations, percentage of live births with 4 to 6 prenatal consultations, and percentage of live births with ≥ 7 prenatal consultations (obtained from SINASC); and socioeconomic variables (Municipal Human Development Index [HDI-M] and GINI index—both obtained using the National Program Human Development Program and illiteracy rate in women aged ≥ 15 years, obtained from DATASUS). Data regarding educational level and income referred to 2010 (i.e., last demographic census), whereas the other variables were obtained from January 2008 to December 2018. Detailed access to databases and calculation of rates and percentages can be consulted in Table 1.

A temporal trend analysis was performed using the GS detection rate in Brazil and its five macro-regions.

We used the Joinpoint Regression program (https://surveillance.cancer.gov/joinpoint/ accessed on 1 November 2021), which verifies annual trends by adjusting a series of trend lines and junction points on a logarithmic scale. The annual percent change (APC) and its respective 95% confidence interval (95%CI) were estimated and tested. The Monte Carlo permutation test assessed the statistical significance and adjusted the best line for each segment. The entire period was analyzed if no changes occurred in trend lines [17]. Pearson’s test analyzed the correlations between GS detection rates and independent variables, using Brazil as the unit of analysis.

Statistical significance was tested using the Pearson product-moment correlation (cor.test function of the R software) [18], which comprised rejection zones for each sample size and significance level. The strength of correlation was classified as weak (r < 0.30), moderate (0.30 < r < 0.70), or strong (r > 0.70) [19,20]. Significance was set at 5%.

## 3. Results

In Brazil, 296,523 cases of GS were reported from 2008 to 2018; the lowest number of GS was reported in 2009 (5265 cases). On the other hand, the highest number of GS was reported in 2018 (59,006 cases). GS detection rate increased by approximately 805%: from 2.49 cases per 1000 live births in 2008 to 20.04 per 1000 live births in 2018. In regions, the country presented disparities in socioeconomic variables and health services (Table 2).

**Figure 1:**
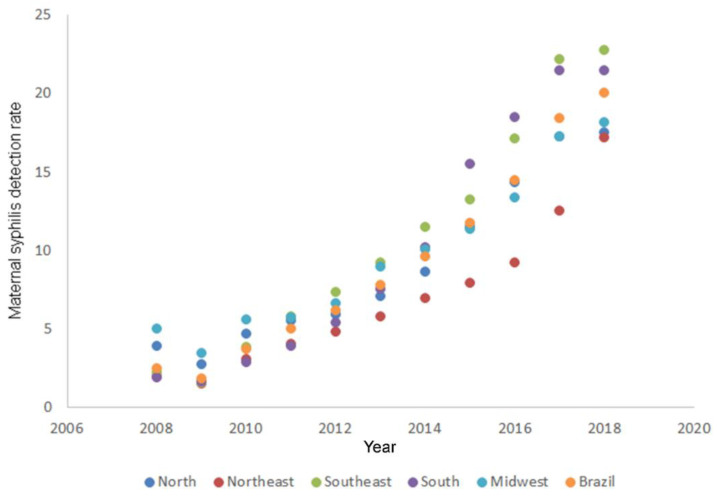
Time series of gestational syphilis detection rate in Brail and all Brazilian regions, according to the Joinpoint model. Brazil, 2008 to 2018. Indicates that the annual percent change is significant.

**Figure 2:**
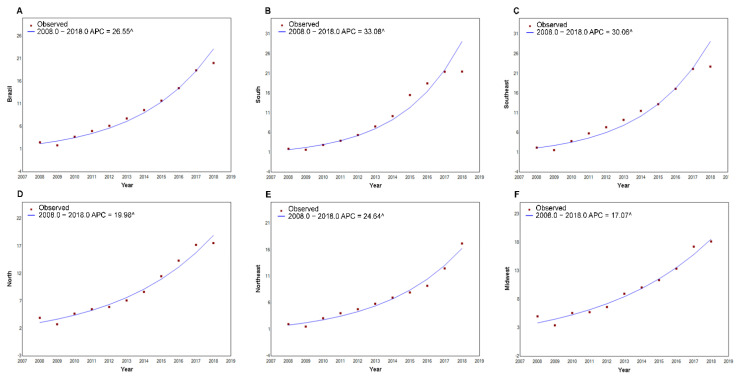
Time series of gestational syphilis detection rate in Brazil (**A**) and by region (South (**B**), Southeast (**C**), North (**D**), Northeast (**E**), and Midwest (**F**)), according to the Joinpoints model. Brazil, 2008 to 2018. ^ Indicates that the annual percent change is significant.

Figure 1 and Figure 2 present the increasing trend in GS detection rate in all Brazilian regions. The Southeast and South had a trend above the national average in recent years. Although the Midwest region was in the third position in 2018 compared with other regions, it presented the lowest APC during the studied period.

A significant increasing trend in the GS detection rate was identified in Brazil (APC = 26.55; 95%CI: 22.1 to 31.2). The South and Midwest regions presented the highest (APC = 33.08; 95%CI: 28.1 to 38.2) and lowest APC (APC = 17.07; 95%CI: 13.6 to 20.6), respectively (Figure 2). The APC in the Southeast (APC = 30.06; 95%CI: 23.5 to 37.0), Northeast (APC = 24.64; 95%CI: 20.4 to 29.0), and North (APC = 19.98; 95%CI: 16.3 to 23.8) indicated an increasing trend with no inflection points during the analyzed period (Figure 1).

HDI-M and illiteracy rate in women aged ≥ 15 years were moderately positively correlated with the GS detection rate (*p* < 0.0001). Variables related to human resources and physical structure in primary health care network (i.e., proportion of primary care physicians, nurses, and primary health care units per inhabitant) showed a moderate negative correlation with GS detection rate (*p* < 0.0001). GS detection rate was also negatively correlated (weak correlation) with the percentage coverage of primary care teams (Table 3).

## 4. Discussion

Our study revealed that all Brazilian regions presented an increasing trend in GS detection rates between 2008 and 2018, and in regions, the country presented disparities in socioeconomic variables and health services, indicating that the syphilis epidemic must be controlled in the country. Despite national policies to combat syphilis (e.g., Rede Cegonha Program in 2011, rapid prenatal syphilis tests in 2012, Strategic Actions Agenda for Reducing Congenital Syphilis in Brazil in 2016, and the No Syphilis Project in 2019) [21,22], studies indicate a growth trend of congenital syphilis in several regions of the country, such as Pará, Amazonia, and Rio de Janeiro [23,24]. Since 2019, the incidence rate of congenital syphilis has declined in the country, probably due to implemented policies [10]. Nevertheless, Brazil is still far from eliminating congenital syphilis.

Although the South region is considered one of the most developed regions in the country, it presents the greatest GS detection rate (average of 33.08% per year). Thus, the increasing trend in GS can also be observed in developed regions, as demonstrated by the moderate positive correlation with HDI-M. Studies conducted in this region indicated limitations and inadequate treatments for GS [25,26] and identified that health professionals had limited knowledge about the prevention and control protocols recommended by the Ministry of Health [27]. The South region also provides easy access to prenatal consultations (rates > 85%) [26,27]; however, this has not been sufficient to prevent the birth of children with syphilis, probably due to the quality of care provided. Moreover, the challenge of treating sexual partners may have aggravated the syphilis epidemic [25,27].

The Southeast region had the highest GS detection rate in 2017 and 2018 and was the second region with the highest increasing trend in the country, despite being the center of economic development in Brazil. The South and Southeast regions account for more than 70% of sectorial production and per capita income and accommodate 56% of the Brazilian population [28]. The São Paulo state, for example, presents large clusters of GS in regions with bad or very bad income, educational level, and health care access [29]. The state of Rio de Janeiro has one of the best Brazilian HDI and good primary health care coverage. However, the incidence rate of congenital syphilis is hard to control, suggesting difficulties in managing GS [24]. Therefore, regions with economic growth and good conditions for human development may still present high GS detection rates, reinforcing the influence of several factors on disease control. Luppi et al. emphasized that the increased access to rapid syphilis tests in campaigns and BHUs in 2017 may have contributed to the increasing trend in detection rates and notifications in the Southeast region [30]. In addition, people from regions with better socioeconomic conditions may have easier access to diagnosis and notification of diseases, increasing detection rates.

Studies have indicated several factors that contribute to increased GS detection rates in the Northeast region, such as high infection rates by *Treponema pallidum* in women exposed to poverty and social vulnerability [31]. Additionally, Conceição et al. showed associations between GS, low educational level, and low purchasing power of women in the Northeast [32]. We observed a positive correlation between GS detection rate and illiteracy rate in women aged ≥ 15 years in Brazil, which may have strongly influenced the growth trend of GS in the Northeast due to the poverty faced by the population from this region. In 2014, the North and Northeast regions accounted for 2.5% and 9.7%, respectively, of financial and insurance activities, indicating regional disparities [28]. Social programs to reduce poverty and eradicate extreme poverty in the Northeast region resulted only in a short-term increase in wages and other remunerations [33]. The lack of investment in the continuing education of primary health care professionals, difficulties in managing resources in the health sector of the Northeast region, and social vulnerabilities faced by the population may have aggravated the syphilis infection [34].

In the North region, studies have shown a delayed diagnosis of GS, inadequate treatments, and untreated sexual partners [23,35]. Access to health facilities was also negatively evaluated, leading to difficulties in prenatal syphilis treatment. The states of Amazonia and Pará presented the worst income, HDI-M, and primary health care coverage of the North region. This region faces limited financial resources from the Ministry of Health, hindering the primary health care network and concentrating primary health care services in urban areas [36]. Our results indicated that the GS detection rate was negatively correlated with physical structure and human resources from primary care. The low concentration of BHU and professionals in rural and riverside areas may also influence the increased GS detection rates in the North region. Therefore, the GS scenario in the North region deserves great attention from public managers because the situation may be more aggravating than the recorded data, mainly due to underreporting caused by difficulties faced by the health system.

Although the Midwest region has a high household income and HDI-M, it presents lower adequacy of prenatal health care, availability of infrastructure, and primary health care coverage than other regions [37]. According to our results, primary health care coverage in Brazil was negatively correlated with the GS detection rate. Social and geographic factors (e.g., cities sharing borders with other countries, large food industries, mining, and important highways) also increase the flow of people and the transmissibility of sexually transmitted diseases [38,39,40].

Difficulties in prenatal health care may also contribute to the increasing trend curve of GS, even in other countries. A study conducted in the United States indicated that the lack of adequate assistance in prenatal health care, the absence of testing, and differences in treatment between regions increased syphilis cases [41]. In this context, measures to improve the quality of prenatal health care in all regions must be implemented to treat GS effectively.

In 2011, the Brazilian Ministry of Health developed a project (*Rede Cegonha*) to organize maternal and child healthcare networks. Access to prenatal health care and tests (including rapid syphilis tests) helped reduce the transmission of syphilis. Committees were also created to investigate the vertical transmission of congenital syphilis, and protocols were published to support practices in health care services for diagnosis and treatment [42]. However, such efforts were insufficient to control the syphilis epidemic. Furthermore, cuts in the public budget, reduced investments in health services, difficulties in the treatment with benzathine penicillin G in the primary health care, and lack of benzathine penicillin G in the world market (especially in 2015) might have influenced the increased GS detection rate in the country [43]. The lack of penicillin G benzathine may have had implications for people infected with syphilis in the decrease in adherence to treatment with other drugs, which may have contributed to an increase in transmissibility of the infection, as well as to the group of pregnant women. The lack of adherence or access to treatment for women infected in the preconception period may also have contributed to the increase in cases in pregnant women.

A positive correlation was identified between GS detection rate, HDI-M, and illiteracy rate in women aged ≥ 15 years. Moreover, a high GS detection rate was observed in cities with high HDI-M, probably due to better health care conditions in BHUs, access to diagnostic methods, and notification systems. Regions with high HDI-M have BHU with better infrastructure and services offered [44], whereas women are less likely to be tested for syphilis in cities with low HDI-M [45]. The BHU infrastructure and services offered differ among Brazilian cities; those in the North and Northeast have greater difficulties coordinating health services and solving population problems than the other regions due to insufficient teams, equipment, and supplies [46]. Our study indicated that cities with women aged ≥ 15 years and highly illiterate presented a high GS detection rate, indicating that low educational level influences the infection by *Treponema pallidum*. This finding corroborates the study of Heringer et al. [24], which showed that social determinants of health contributed to GS infection. Despite the contradiction presented in the results of our study with the HDI and the illiteracy rate in women aged 15 years or more in correlation with the rate of gestational syphilis, the scientific literature confirms the existence of a correlation between high HDI and higher rates of detection of gestational syphilis, as well as low education with higher rates of syphilis in pregnant women. The educational variable analyzed in isolation could present a statistical result different from the HDI correlation, which, although it is an index composed of education indicators (literacy and enrollment rate), longevity (life expectancy at birth), and income (GDP per capita).

The results showed that cities with a low proportion of professionals per inhabitant in primary health care and BHU have more cases of GS, revealing the importance of human resources and infrastructure to control syphilis. However, primary health care faces difficulties establishing medical doctors due to the poor distribution of these professionals in Brazil [47].

GS detection rate was also negatively correlated with the proportion of primary health care coverage. Therefore, the number of GS was high in cities with low coverage. In contrast, studies have demonstrated a positive association between primary health care coverage and syphilis cases due to improvements in notifications, implementation of rapid tests, and training of professionals [44,46]. In agreement with our study, Nunes et al. showed a negative association between primary health care coverage and the GS detection rate [42]. Low primary health care coverage may reduce educational actions to prevent diseases and increase difficulties accessing BHU professionals, attending prenatal consultations, and receiving effective treatment. Studies have also indicated that better access to prenatal consultations decreases GS detection rates [31].

Incentive programs for establishing primary health care professionals in areas of difficult access, valorization of training and career, and financial incentive to expand and better redistribute BHU may facilitate access, guarantee diagnosis and effective treatments, and improve important health promotion actions to control syphilis. Primary health care coverage is essential to reducing GS and increasing prenatal consultations.

In the country, the most commonly used non-treponemal test is the Venereal Disease Research Laboratory (VDRL) [48]; however, the Rapid Test Reagin (RTR) may also be available [49,50]. The most frequently performed treponemal test is the Rapid Test (RT), after the institution of the ordinance that regulated its financing and implementation in routine prenatal care [51]; other tests can also be found, such as the Treponema pallidum Hemagglutination Test (TPHA); Fluorescent treponemal antibody absorption (FTA-abs); and Enzyme-linked immunosorbent assay (ELISA) [49,50]. Access to the types of tests can differ between municipalities [50], making the uniformity of the tests performed a limitation in the dataset available for consultation. Since the data for obtaining the detection rates of gestational syphilis came from SINAN, it is not possible to identify in the system the test used in each municipality for the diagnosis of gestational syphilis.

This study is not free of limitations. The trend analysis and correlation were limited to the interval between 2008 and 2018 because data regarding the number of GS cases were available in SINAN only until 2018. Thus, possible underreporting may represent an obstacle to more reliable information regarding the incidence of cases. Future studies analyzing the correlations between GS detection rates (stratified by region) and the independent variables adopted in this study are essential for identifying possible regional disparities.

The strong points of the research were inserted into the discussion as follows: This is the first approximation in the literature that demonstrates the correlation of the detection rate of gestational syphilis with the proportion of physicians, nurses, and basic health units per inhabitants, from a study encompassing all municipalities in Brazil, highlighting the need to strengthen primary health care to combat syphilis. Based on the method used in the research, it is possible to guide health surveillance actions at the national and municipal levels in the implementation of joint actions with other sectors so that the detection rate of gestational syphilis is reduced and the factors related to it are resolved.

## 5. Conclusions

In conclusion, the GS detection rate increased in all Brazilian regions from 2008 to 2018 and was influenced by socioeconomic and health care indicators. The South region presented the highest APC, whereas the Midwest region had the lowest APC compared with the other regions. GS detection rate was negatively correlated with primary health care coverage, newborns with four to six prenatal consultations, and the proportion of nurses, doctors, and BHU per inhabitant in the primary health care.

We also observed a positive correlation between the GS detection rate and HDI-M and the illiteracy rate among women. The time series analysis of GS in Brazilian territory may contribute to public policies against the syphilis epidemic in the country and support health systems in other countries to control the transmissibility of the disease.

In conclusion, the GS detection rate increased in all Brazilian regions from 2008 to 2018. The south region had the highest APC, while the Midwest region had the lowest APC compared to the other regions. The GS detection rate showed significant correlations with socioeconomic and health indicators.

The positive correlation between the GS detection rate and the HDI and the illiteracy rate among women, which, although contradictory since the HDI is also an index composed of education components, can be related to the increase in the offer of tests and access to health services in more developed municipalities. Schooling, analyzed separately, points to the importance of access to these women’s knowledge to prevent infection. Correlations with health indicators show the importance of structuring the PHC with adequate numbers of professionals and health units, improvement in PHC coverage, and greater access to prenatal consultations to reduce the GS detection rate.

It is important to emphasize that variations in Brazilian government protocols for screening syphilis may imply differences in diagnostic procedures among professionals. The recommendation to perform only one test in pregnant women as a diagnostic criterion may contribute to an increase in the number of false positives, culminating in an increase in the GS detection rate and an increase in the trend curve. Improving access to and funding for treponemal and non-treponemal tests, establishing a gold standard test, and implementing measures that strengthen prevention and protection against syphilis are essential for controlling infection in the country.

The analysis of GS time series in Brazilian territory can contribute to public policies to face the syphilis epidemic in the country and support health systems in other countries to control the transmissibility of the disease.

## Figures and Tables

**Table 1:** Indicators of gestational syphilis detection rate, health care, and socioeconomic and health conditions. Brazilian cities, 2008 to 2018.

Outcomes	Concept	Data Source
GS detection rate	Number of notified cases of GS, divided by total live births in a determined time and place, and multiplied by 1000.	SINAN, Information System of Live Births Number of notified cases of gestational syphilis. DATASUS → Health information (Tabnet) → Epidemiological and morbidity → Notifiable diseases and conditions—from 2007 onwards (SINAN) → gestational syphilis → Brazil by city → City of residence → Year of diagnosis → Period from 2008 to 2018 http://tabnet.datasus.gov.br/cgi/tabcgi.exe?sinannet/cnv/sifilisgestantebr.def Accessed on 25 June 2021 Number of total live births DATASUS → Health information → Vital statistics → Live births—from 1994 to 2019 → Live births → Births per residence/mother http://tabnet.datasus.gov.br/cgi/tabcgi.exe?sinasc/cnv/nvbr.def Accessed on 25 June 2021
HDI-M	Average income, education, and longevity.	PNUD AtlasBrasil → Collection → Library → Database → Demographic census 2010 https://onedrive.live.com/?authkey=%21ABiV0mb1HeyuOxU&cid=124653557C0404EC&id=124653557C0404EC%2123017&parId=124653557C0404EC%2122899&action=locate Accessed on 29 June 2021
GINI Index	Measures the degree of inequality in the distribution of individuals according to household income per capita. Zero represents no inequality, whereas 1 indicates inequality. Limited to people who live in permanent private households.	PNUD AtlasBrasil → Collection → Library → Database → Demographic census 2010 https://onedrive.live.com/?authkey=%21ABiV0mb1HeyuOxU&cid=124653557C0404EC&id=124653557C0404EC%2123017&parId=124653557C0404EC%2122899&action=locate Accessed on 29 June 2021
Illiteracy rate in women aged ≥ 15 years	Percentage of people aged ≥ 15 years who cannot read and write a simple note in a known language, considering the total number of residents of same age in a given geographic space and period.	DATASUS DATASUS → Health information → Demographic and socioeconomic → Educational level → Education of population aged 15 and over → Brazil by city → Women → period 2010 http://tabnet.datasus.gov.br/cgi/tabcgi.exe?ibge/censo/cnv/alfbr.def Accessed on 29 July 2021
Proportion of doctors per inhabitant in the primary health care	Number of specific professionals in a given location and period, divided by the population of a specific location and period, and multiplied by 3500. Note: 3500 is the maximum number of people that a professional must cover according to the primary health care ordinance 2436/2017	CNES DATASUS → Health information → Assistance network → CNES—Human resources from August 2007—Occupations classified by CBO 2002 Professionals → Doctor of Family Health Strategy, Doctor of Family and Community → Attend on SUS → December 2008, December 2009, December 2010, December 2011, December 2012, December 2013, December 2014, December 2015, December 2016, December 2017, December 2018 http://tabnet.datasus.gov.br/cgi/tabcgi.exe?cnes/cnv/prid02br.def Accessed on 28 June 2021
Proportion of nurses per inhabitant in primary health care	Number of specific professionals in a given location and period, divided by the population of a specific location and period, and multiplied by 3500. Note: 3500 is the maximum number of people that a professional must cover according to the primary health care ordinance 2436/2017	CNES DATASUS → Health information → Assistance network → CNES-Human resources from August 2007—Occupations classified by CBO 2002 → Professionals → Occupation with graduation → Nurse of the Family Health Strategy, Nurse of Family Health → December 2008, December 2009, December 2010, December 2011, December 2012, December 2013, December 2014, December 2015, December 2016, December 2017, December 2018 http://tabnet.datasus.gov.br/cgi/tabcgi.exe?cnes/cnv/prid02br.def Accessed on 2 July 2021
Proportion of health units per inhabitant in primary health care	Number of health units + number of family health units + number of health centers in a given place and period, divided by the population of that place and period, and multiplied by 14,000. Note: Each health unit can absorb up to four teams to maintain effectiveness, according to the primary health care ordinance 2436/2017	CNES DATASUS → Health information → Assistance network → Types of establishment → Type of establishment: Health center/health unit, health center, family health unit. Period: December 2008, December 2009, December 2010, December 2011, December 2012, December 2013, December 2014, December 2015, December 2016, December 2017, December 2018 http://tabnet.datasus.gov.br/cgi/tabcgi.exe?cnes/cnv/estabbr.def Accessed on 2 July 2021
Percentage of coverage of the primary health care team	Estimated population coverage in primary health care (i.e., the percentage of population covered by family health strategy teams and equivalent traditional primary health care teams, normalized by the population estimative)	e-Gestor AB Primary care coverage → Options → Geographic unit by period → Cities → All regions, all states, all cities → Competence December 2008, December 2009, December 2010, December 2011, December 2012, December 2013, December 2014, December 2015, December 2016, December 2017, December 2018. https://egestorab.saude.gov.br/paginas/acessoPublico/relatorios/relHistoricoCoberturaAB.xhtml Accessed on 26 June 2021
Percentage of live births with no prenatal consultation	Number of live births with no prenatal consultation from a given location and period, divided by the number of total live births from the same location and period, and multiplied by 100.	SINASC DATASUS → Health information → Vital statistics → Live births—from 1994 to 2019 → No prenatal consultation → Birth by mother, by city, and by year of birth → Period from 2008 to 2018 http://tabnet.datasus.gov.br/cgi/tabcgi.exe?sinasc/cnv/nvbr.def Accessed on 2 July 2021
Percentage of live births with one to three prenatal consultations	Number of live births with one to three prenatal consultations from a given location and period, divided by the number of total live births from the same location and period, and multiplied by 100.	SINASC DATASUS → Health information → Vital statistics → Live births—from 1994 to 2019 → One to three prenatal consultations → Birth by mother, by city, and by year of birth → Period from 2008 to 2018 http://tabnet.datasus.gov.br/cgi/tabcgi.exe?sinasc/cnv/nvbr.def Accessed on 2 July 2021
Percentage of live births with four or more prenatal consultations	Number of live births with four or more prenatal consultations from a given location and period, divided by the number of total live births from the same location and period, and multiplied by 100.	SINASC DATASUS → Health information → Vital statistics → Live births—from 1994 to 2019 → Four to six prenatal consultations and seven or more prenatal consultations → Birth by mother, by city, and by year of birth → Period from 2008 to 2018 http://tabnet.datasus.gov.br/cgi/tabcgi.exe?sinasc/cnv/nvbr.def Accessed on 29 July 2021
Percentage of live births with seven or more prenatal consultations	Number of live births with seven or more prenatal consultations from a given location and period, divided by the number of total live births from the same location and period, and multiplied by 100.	SINASC DATASUS → Health information → Vital statistics → Live births—from 1994 to 2019 → Four to six prenatal consultations and seven or more prenatal consultations → Birth by mother, by city, and by year of birth → Period from 2008 to 2018 http://tabnet.datasus.gov.br/cgi/tabcgi.exe?sinasc/cnv/nvbr.def Accessed on 2 July 2021

HDI-M: Municipal Human Development Index; DATASUS: Department of Informatics of the Unified Health System; SINAN: Notifiable Diseases Information System; PNUD: United Nations Development Program; SINASC: Live Birth Information System; CNES: National Registry of Health Establishments; CBO: Brazilian Classification Occupation Classification; e-Gestor AB: e-Manager primary care.

**Table 2:** Gestational syphilis detection rate, socioeconomic variables, and health services from 2008 and 2018, divided by Brazilian region.

	Year	Northeast	North	Midwest	South	Southeast	Brazil
OUTCOMES Gestational syphilis detection rate ^a^							
	2008	1.98	3.92	4.99	1.88	2.19	2.49
	2018	17.19	17.54	18.14	21.47	22.73	20.04
OUTCOMES HDI-M ^b^							
	2010	0.6510	0.6564	0.7449	0.7508	0.7594	0.724
OUTCOMES GINI ^c^							
	2010	0.5555	0.5781	0.5389	0.4874	0.5220	0.5322
OUTCOMES Illiteracy rate in women aged > 15 years ^d^							
	2010	22.9045	11.1934	12.0883	8.8289	8.8270	13.0334
OUTCOMES Percentage of primary health care coverage ^e^							
	2008	90.9014	72.8213	88.6699	83.1240	79.4361	84.1578
	2018	96.5358	87.7937	93.1030	91.8167	88.7517	92.2017
OUTCOMES Proportion of doctors in primary health care per inhabitants ^f^							
	2008	0.4020	0.2861	0.2614	0.2698	0.2415	0.2955
	2018	0.5215	0.4473	0.4510	0.4969	0.4004	0.4551
OUTCOMES Proportion of nurses in primary health care per inhabitants ^g^							
	2008 2018	0.7391 0.9021	0.5559 0.6651	0.5443 0.6724	0.5803 0.7326	0.4231 0.5845	0.5537 0.7059
OUTCOMES Proportion of BHU in primary health care per inhabitants ^h^							
	2008	4.0532	3.3692	2.9848	3.4804	2.2160	3.0644
	2018	4.4228	3.1581	2.8296	3.3620	2.2188	3.1108
OUTCOMES Percentage of newborns with no prenatal consultation ^i^							
	2008	2.0568	4.3783	1.2571	1.0719	1.2143	1.8016
	2018	2.1175	3.7860	1.2113	0.9344	1.0969	1.6660
OUTCOMES Percentage of newborns with one to three prenatal consultation ^j^							
	2008	10.5624	15.9385	5.7654	4.4991	4.5661	7.7112
	2018	6.6496	12.2511	5.7075	3.3251	3.9109	5.6640
OUTCOMES Percentage of newborns with four or more consultation ^l^							
	2008	87.3808	79.6834	92.9775	94.4290	86.8963	90.4872
	2018	91.2329	83.9629	93.0812	95.7405	87.7669	92.6700

^a^ GS detection rate was obtained by dividing the number of reported cases in a given region and period by the number of live births in a given region and period, multiplied by 1000. ^b,c^ Sum of the index per municipality multiplied by its respective population, divided by the sum of the populations of the municipalities. ^d^ Sum of the product of the index by its female population divided by the sum of female populations. ^e^ Sum of the index per municipality multiplied by its respective population divided by the sum of the populations of the municipalities. ^f,g,h^ Sum of the number of physicians/nurses/BHU within each region divided by the total population of each region multiplied by 3500 to the proportion of professionals and by 14,000 for the proportion of BHU. ^i,j,l^ Sum of the number of each consultation divided by the total number of live births in its respective region.

**Table 3:** Correlations between gestational syphilis detection rate and socioeconomic and health care indicators.

Outcomes	Correlation Coefficient	*p*
GINI Index	−0.0090	0.8431
HDI-M	0.3168	<0.0001
Illiteracy rate in women aged > 15 years	0.3466	<0.0001
Percentage of newborns with no prenatal consultation	0.0029	0.9487
Percentage of newborns with one to three prenatal consultations	0.0539	0.2373
Percentage of newborns with four or more prenatal consultations	−0.0517	0.2572
Percentage of primary health care coverage	−0.2304	<0.0001
Proportion of doctors in primary health care per inhabitant	−0.3127	<0.0001
Proportion of nurses in primary health care per inhabitant	−0.3911	<0.0001
Proportion of BHU in primary health care per inhabitant	−0.3842	<0.0001

## Data Availability

The data presented in this study are available on request from the corresponding author.
